# A comparative analysis of open heart surgery and minimally invasive cardiac surgery in exercise-based cardiac rehabilitation

**DOI:** 10.1186/s13019-024-02871-z

**Published:** 2024-06-27

**Authors:** Maciej Marek Hubisz, Jan Gerrit van der Stouwe, Mira Ziob, Sonja Steiner, Neslihan Uzun, Sandra Weibel, Vlada Lesan, Dominic Erni, Ladina Meier-Ruge, Hector Rodriguez Cetina Biefer, Omer Dzemali, Jan Vontobel, David Niederseer

**Affiliations:** 1 Hochgebirgsklinik, Medicine Campus Davos, Herman-Burchard-Strasse 1, 7270 Davos, Switzerland; 2 Department of Cardiology, University Hospital Basel, Basel, Switzerland; 3 Department of Cardiac Surgery, University Heart Center Zurich, University Hospital Zurich, University of Zurich, Zurich, Switzerland; 4 Department of Cardiac Surgery, City Hospital of Zurich Triemli, Zurich, Switzerland; 5 Department of Cardiology, Center of Translational and Experimental Cardiology (CTEC), University Heart Center Zurich, University Hospital Zurich, University of Zurich, Zurich, Switzerland; 6 Christine Kühne Center for Allergy Research and Education (CK-CARE), Medicine Campus Davos, Davos, Switzerland

**Keywords:** Inpatient exercise-based cardiac rehabilitation, Open heart surgery, Minimally invasive cardiac surgery

## Abstract

**Background:**

Historically, the majority of patients admitted to inpatient exercise-based cardiac rehabilitation (EBCR) have undergone open heart surgery (OHS). However, with advances in minimally invasive cardiac surgery (MICS), these patient groups are also increasingly referred for inpatient EBCR. Herein, we aimed to compare the progress of these groups during rehabilitation.

**Methods:**

In this prospective, nonrandomized study, 403 inpatient EBCR patients were recruited from December 2022 until September 2023 and stratified into two groups: OHS, and MICS. Participants completed a 3-4-week certified EBCR program. The primary endpoint was defined as a change in the 6-minute walk test (6MWT). Moreover, a comprehensive panel of quality-of-life (QoL) assessments were performed at admission and discharge.

**Results:**

At baseline, patients with OHS were older (66 years [IQR 59 – 72]), more often male (83%), and underwent emergency/urgent procedures more often (20%) than patients with MICS. Furthermore, patients with MICS showed a better 6MWT at admission (426 meters [IQR 336 – 483]) compared to patients with OHS (381 meters [IQR 299 – 453]). While all patients were able to increase the distance in the 6MWT, regression analyses in fully adjusted models showed no difference in improvements between the two groups (β -5, 95% CI, -26 – 14, *p* = 0.58). Moreover, during EBCR, we observed significant improvements in all QoL measures in all groups.

**Conclusions:**

In this study, improvements in fitness, as assessed by the 6WMT were observed in all groups. Furthermore, multiple QoL measures improved equally across all groups. These encouraging results emphasize the importance of EBCR.

## Background

Exercise-based cardiac rehabilitation (EBCR) is an essential pilar for patients with coronary and valvular heart disease to improve outcomes after surgery or interventions [1–4]. Historically, the majority of patients referred for inpatient EBCR have undergone open heart surgery (OHS), but advances in minimally invasive cardiac surgery (MICS) have led to increased admissions of these patients to inpatient EBCR [5]. Individualized programs are created for each patient taking into consideration symptoms, comorbidities, age, and fitness.

Previous studies have shown that exercise capacity, measured by the 6-minute walking test (6MWT), can be improved equally among all types of OHS [6]. However, data on the comparison between OHS and MICS are lacking. Furthermore, EBCR not only focuses on exercise but also takes a holistic approach, including diet/nutritional counseling, weight control management, lipid management, blood pressure (BP) monitoring and management, smoking cessation, and psychosocial management [7]. Although the importance of cardiovascular risk management has been extensively documented [8], recent studies have shed light on the importance of treating of psychological distress during EBCR among patients with cardiac disease [9]. However, comparisons between patients after OHS and MICS are lacking to date.

Herein, we aimed to close these knowledge gaps and compare improvements in fitness as well as psychological parameters in patients after OHS and MICS during inpatient EBCR.

## Methods

### Study design, participants and material

This is a prospective cohort study, that included 403 patients who were referred for inpatient EBCR at the Hochgebirgsklinik Davos, between December 2022 and September 2023. Patients were stratified into two cohorts that included OHS (*n*=300) and MICS (*n*=103. OHS was defined as surgery using a total sternotomy. MICS included patients who underwent surgery either by partial sternotomy or thoracotomy. Participants had to be over the age of 18 years and patients with active malignancy and the use of left ventricular assist devices were excluded. All patients underwent an individualized inpatient EBCR program certified by the European Association for Preventive Cardiology [10] as well as by the Swiss SCPRS (Swiss Working Group for Cardiovascular Prevention, Rehabilitation and Sports Cardiology) Society for an average duration of 3-4 weeks. This typically included the following weekly activities: 6 hours of walks, weight training, cycle ergometer training, and 2.5 hours of gymnastics training each. More specifically, walk-sessions were adapted to three different endurance levels. The most fit group undertook a continuous 60-minute walk without any pauses. The intermediate fitness group completed a 60-minute walk, interspersed with three brief pauses of 1-2 minutes each, suitable for those requiring slight rest. The least fit group participated in a 30-minute indoor walking training with pauses as needed, catering to the needs of the frailest patients. Weight training was customized according to the type of surgery patients had undergone. Those who had OHS performed exercised using only 10% of their body weight, focusing on symmetrical loads to ensure balanced muscular engagement. Patients who underwent MICS were allowed to train with normal loads, facing no restrictions and thus enabling a more intensive strength training session after resolution of post-surgical hematoma. Special considerations were made for patients with pacemakers; exercises were modified to avoid full loading or raising of the limb on the side of the pacemaker implantation beyond 90 degrees, ensuring a safe workout environment. Gymnastics sessions were held in a training hall and included a mix of coordination exercises, balance training, and light games.

Patients received at least one consultation with a dietician. Those diagnosed with type 2 diabetes mellitus receiving a continuous glucose monitor to aid in nutrition and glucose level management. All meals were based on the Mediterranean diet and were provided as per guideline recommendations to reduce cardiovascular risk. Additionally, patients with diabetes or heart failure had their medication regimens reviewed and adjusted weekly. A specialist heart failure nursing team was involved in a structured and intensive educational intervention with every heart failure patient, involving long term planning, as well as integrating additional care givers or the family members. Each patient had a Wound Counselling prescribed as directed by the surgeon performing the procedure. Physiotherapists, ergotherapists as well as activation therapist were individually involved in the CR program, reflecting the particular needs of our most frail patients. Patients with a subjective psychological burden were provided with cardiopsychological care at an intensity individually tailored to the patient's needs. Data on age, sex, BMI, heart failure, cardiovascular risk factors, coronary artery disease, rhythm disorders, left ventricular ejection fraction (LVEF), medication, social status, employment, type and timing of procedure, BP, and heart rate (HR) were collected at admission. Furthermore, a blood sample was taken to evaluate leukocyte, hemoglobin, creatinine, and C-reactive protein (CRP) levels. Surface 12-lead electrocardiograms (ECGs) were obtained at admission and discharge. All patients completed the 6MWT as well as a timed up and go test (TUG) and a comprehensive panel of quality of life (QoL) assessments including the functional independence measure (FIM), MacNew Heart (MNH) questionnaire, and the Hospital Anxiety and Depression Scale (HADS), which is divided into the HADS-Depression (HADS-D), HADS-Anxiety (HADS-A), and HADS-Total (HADS-T) at admission and discharge. Furthermore, all patients completed a standardized exercise test at discharge. All participants provided written informed consent. The study was conducted in accordance with the Declaration of Helsinki after approval by the local ethics committee.

Every patient undergoing OHS or MICS was routinely referred to the ECBR program. Exceptions to this referral are rare and typically occur only at the patient's request or if the patient is fit enough to be followed in an outpatient regiment. Therefore, there is a selection bias, as the inpatient program involves a frailer patient group. At the time of the survey, the cost of cardiac rehabilitation was CHF 614.5 per patient per day. Most of these costs are covered by health insurance, with patients paying out of pocket only in exceptional circumstances.

### Statistical analysis

Baseline characteristics were stratified by type of intervention (OHS or MICS). Continuous variables are presented as mean and standard deviations (SDs) or median and interquartile ranges (IQRs), and are compared using Mann-Whitney-U-test or Fischer’s exact test, as appropriate. Categorical variables are presented as counts and percentages (%) and were compared using the chi-square test.

The primary endpoint was defined as the change in walking distance in the 6MWT from admission to discharge between the two groups. The secondary endpoints were changes in the FIM, HADS, MNH, and TUG scores from admission to discharge between the two groups. Additionally, in order to identify patients who might benefit more from rehabilitation, patients within each group (OHS and MICS) were stratified by age (under and above and 60 years of age) and type of surgery (revascularization only and valve surgery only). Analysis of covariance (ANCOVA) was used to assess these changes between admission and discharge. In Model 1, the primary and secondary endpoints were corrected for their baseline values at admission. Model 2 included the respective baseline value at admission as well as well as sex (categorical) and age (continuous). Model 3 was additionally corrected for fitness at discharge, defined as the maximum watt in percent of the calculated maximum (continuous). Finally, Model 4 included all variables from Model 3 and was additionally corrected for heart failure and change in hemoglobin from admission to discharge. Complete data for all variables used in the ANCOVA were available. Statistical significance for the primary analysis of the 6MWT was established by two-sided P values <0.05. For all secondary endpoints, multiple testing was considered, and after Bonferroni correction, a two-sided P value of <0.01 established statistical significance. Sample sizes were calculated using a standard two-sided significance level (α) of 0.05 and power of 0.80 assuming a clinically relevant effect size of 0.3 as well as an uneven distribution in the groups since patients with OHS were expected to make up the largest portion of patients. All analyses were performed in SPSS version 26 (IBM, Armonk, NY, USA).

## Results

### Baseline characteristics

Baseline characteristics of patients stratified into the two cohorts by type of procedure are shown in Table 1. Median inpatient hospital stay was similar across all groups and between 20 and 22 days. Patients with OHS were older (66 years [IQR 59 – 72]) and has CAD more often (65%) compared to patients after MICS (63 years [IQR 55 – 59], 20% CAD). However, MI was similar between both groups. Accordingly, cardiovascular risk factors were significantly less common in patients after MICS (hypertension 47%, type 2 diabetes mellitus 6%, dyslipidemia 50%) compared to patients after OHS (hypertension 64%, type 2 diabetes mellitus 19%, dyslipidemia 67%). Consequently, the use of aspirin, P2Y12 inhibitors, and statins in patients after MICS (55%, 11%, and 47%, respectively) was significantly lower compared to patients after OHS (82%, 27%, and 71%, respectively) There were no differences with regard to heart failure, LVEF, family status, employment status, or living area.

**Table 1:** Baseline characteristics

	**Open heart surgery** ***n* = 300**	**Minimally invasive surgery** ***n* = 103**	***p* value**
**Patient characteristics**			
Rehabilitation duration (days)	22 (20 – 27)	20 (19 – 26)	0.08
Time between surgery and admission (days)	8 (7 – 9)	8 (7 – 9)	0.52
Age (years)	66 (59 – 72)	63 (55 – 59)	0.01
Male	248 (83)	77 (75)	0.05
Body mass index (kg/m^2^)	26.4 (23.4 – 29.1)	24.7 (22.6 – 27.8)	0.01
**Heart failure (any)**	74 (25)	24 (23)	0.78
HFpEF	4 (1)	1 (1)	0.77
HFmrEF	46 (15)	15 (15)	0.85
HFrEF	20 (7)	3 (3)	0.16
HFimpEF	4 (1)	5 (5)	0.04
**Myocardial Infarction (total)**	30 (10)	5 (5)	0.08
STEMI	7 (2)	2 (2)	–
NSTE-ACS	20 (7)	3 (3)	–
Unknown	3 (1)	0 (0)	–
**Coronary artery disease (total)**	169 (65)	20 (20)	<0.001
Single-vessel	15 (5)	10 (10)	–
Multivessel (2)	40 (13)	7 (7)	–
Multivessel (3)	135 (45)	3 (3)	–
Unknown	6 (2)	0 (0)	–
**Cardiac arrest**			
Out-Of-Hospital	2 (1)	0 (0)	–
In-Hospital	3 (1)	0 (0)	–
**Atrial fibrillation/flutter**			
Postoperative	53 (18)	26 (25)	0.07
Preoperative paroxysmal	20 (6)	9 (9)	–
Preoperative persistent	8 (2)	1 (1)	–
Preoperative permanent	3 (1)	0 (0)	–
**Cardiovascular risk factors**			
Obstructive sleep apnea	14 (5)	3 (3)	–
Smoking	202 (67)	75 (73)	0.86
pack years	10 (±1)	8 (±2)	0.41
Hypertension	192 (64)	48 (47)	0.03
Type 2 diabetes mellitus	58 (19)	6 (6)	0.01
Obesity and overweight	100 (33)	31 (30)	0.14
Dyslipidemia	200 (67)	51 (50)	0.01
CKD (KDIGO G3a or higher)	10 (3)	3 (3)	0.34
Peripheral artery disease	22 (7)	2 (2)	–
**Electrocardiogram**			
Left bundle branch block	16 (5)	3 (3)	0.42
Richt bundle branch block	53 (18)	22 (21)	0.47
**Left ventricular ejection fraction**	55 (50 – 60)	55 (49 – 58)	0.32
**Medication**			
Aspirin	246 (82)	57 (55)	<0.001
P2Y12-Inhibitor	80 (27)	11 (11)	<0.001
DOAC	23 (8)	8 (8)	0.44
Vitamin K antagonist	64 (21)	42 (41)	<0.001
ACE/ARB	151 (50)	51 (50)	0.53
ARNI	4 (1)	2 (2)	0.67
Beta blocker	221 (74)	69 (67)	0.21
Digoxin	0 (0)	0 (0)	
Loop diuretics	74 (25)	31 (30)	0.37
Thiazide	10 (3)	7(7)	0.03
SGLT2i	49 (16)	12 (12)	0.02
MRA	43 (14)	14 (14)	0.52
Antiarrhythmics	31 (10)	18 (18)	0.12
Calcium channel blocker	53 (18)	12 (12)	0.23
Statin	213 (71)	48 (47)	<0.001
Ezetimibe	61 (20)	4 (4)	0.002
Oral antidiabetics	49 (16)	6 (6)	0.02
Insulin	17 (6)	1 (1)	0.14
GPL-1 RA	7 (2)	2 (2)	0.40
**Family status**			
Married	182 (61)	55 (53)	0.02
Single	33 (11)	14 (14)	0.66
Divorced	43 (14)	14 (14)	0.72
Widowed	6 (2)	4 (4)	0.24
Informal	69 (8)	11 (11)	0.62
**Employment**			
While collar	154 (51)	59 (57)	0.10
blue collar	120 (40)	37 (36)	0.09
retired	153 (51)	48 (47)	0.18
**Living areas**			
Urban	138 (46)	49 (48)	0.78
Rural	157 (52)	51 (50)	0.67
**Procedural characteristics**			
**Timing of procedure (total)**			
Emergency/Urgent	60 (20)	8 (8)	0.001
Elective	244 (81)	97 (97)	0.002
**Revascularization (total)**	200 (67)	15 (15)	<0.001
Emergency/Urgent	51 (17)	6 (6)	–
Elective	149 (50)	9 (9)	–
**Valvular procedure (total)**	152 (51)	65 (63)	0.01
Aortic (total)	105 (35)	34 (34)	–
Stenosis	58 (19)	15 (15)	–
Regurgitation	47 (16)	19 (19)	–
Mitral (total)	42 (14)	46 (45)	–
Stenosis	1 (0)	1 (1)	–
Regurgitation	41 (14)	45 (45)	–
Tricuspid regurgitation	5 (2)	2 (2)	–
**Aortic procedure (total)**	85 (28)	16 (16)	0.001
Emergency/Urgent	16 (5)	4 (4)	–
remodeling	1 (0)	0 (0)	–
root replacement	3 (1)	2 (2)	–
ascending replacement	6 (2)	1 (1)	–
hemiarch replacement	6 (2)	0 (0)	–
total arch replacement	0 (0)	1 (1)	–
Elective	69 (23)	12 (12)	–
remodeling	1 (0)	1 (1)	–
root replacement	16 (5)	1 (1)	–
ascending replacement	29 (10)	6 (6)	–
hemiarch replacement	22 (7)	4 (4)	–
AAA repair	1 (0)	0 (0)	–

Data are shown as mean and standard deviation or median and interquartile range if skewed. Categorical data are shown as numbers and percentages. p values are based on Kruskal Wallis test, ANOVA, chi square tests, or Fischer’s exact test as appropriate

*Abbreviations: AAA* Abdominal aortic aneurysm, *ACE* angiotensin-converting-enzyme, *ARB* angiotensin receptor blocker, *ARNI* Angiotensin receptor neprilysin inhibitor, *BMI* body mass index, *CKD* chronic kidney disease, *DOAC* direct oral anticoagulant, GLP-1 RA = GLP-1 receptor agonist, *HFimpEF* Heart failure with improved ejection fraction, *HFmrEF* Heart failure with mildly reduced ejection fraction, *HFpEF* Heart failure with preserved ejection fraction, *HFrEF* Heart failure with reduced ejection fraction, *MRA* Mineralocorticoid Receptor Antagonists, *NSTE-ACS* Non-ST-elevation acute coronary syndrome, *SGLT2i* Sodium-glucose cotransporter-2, *STEMI* ST-elevation myocardial infarction

Emergency/urgent procedures were more common in patients who received OHS (20%) compared to patients who received MICS (8%). Revascularization was common in patients who received OHS (67%) and rare in patients with MICS (15%). Accordingly, valvular procedures were most common in MICS (63%) and less common in OHS (51%) Overall, 28% of patients with OHS underwent aortic procedures, comprising 93% of all aortic procedures in this cohort. There was some overlap between the type of procedure, since many patients with revascularization also underwent a valvular procedure. Additionally, there was some overlap between emergency/urgent and elective procedures since some patients underwent an elective procedure after an initial emergency/urgent procedure.

### Laboratory parameters

At admission, patients after OHS had higher leukocyte (10.0 G/l [IQR 8.1 – 11.9] compared to patients after MICS (9.0 G/l [IQR 7.3 – 11.3]), lower hemoglobin (109 g/l [IQR 99 – 120], and 117 g/l [IQR 106 – 132], respectively), and similar C-reactive protein (CRP) (36 mg/l [IQR 23 – 66], and 43 mg/l [IQR 26 – 71], respectively) levels. During rehabilitation, the leukocyte and CRP levels decreased, and hemoglobin increased in all groups (Table 2).

**Table 2:** Changes in the 6-minute walk test score, quality of life measurements, vital signs, and laboratory parameters from admission to discharge

	**Open heart surgery** ***n* = 300**	**Minimally invasive surgery** ***n* = 103**	***p* value**
**Scores**			
6MWT (meters)			
Admission	381 (299 – 453)	426 (336 – 483)	0.04
Discharge	525 (450 – 589)	578 (476 – 667)	<0.001
*p value (A vs. D)*	<0.001 *(n=266)*	<0.001 *(n=93)*	
FIM			
Admission	102 (90 – 112)	108 (97 – 116)	0.01
Discharge	124 (119 – 126)	125 (121 – 126)	0.02
*p value (A vs. D)*	<0.001 *(n=289)*	<0.001 *(n=101)*	
HADS A			
Admission	2 (1 – 5)	3 (1 – 6)	0.65
Discharge	1 (0 – 3)	1 (0 – 2)	0.66
*p value (A vs. D)*	<0.001* (n=214)*	<0.001 *(n=76)*	
HADS D			
Admission	3 (1 – 5)	2 (1 – 5)	0.36
Discharge	1 (0 – 2)	1 (0 – 2)	0.69
*p value (A vs. D)*	<0.001* (n=214)*	<0.001 *(n=76)*	
HADS T			
Admission	5 (2 – 10)	5 (2 – 10)	0.84
Discharge	2 (1 – 5)	2 (0 – 4)	0.93
*p value (A vs. D)*	<0.001* (n=214)*	<0.001 *(n=76)*	
MNH			
Admission	135 (121 – 149)	138 (125 – 152)	0.30
Discharge	160 (149 – 169)	165 (156 – 173)	0.003
*p value (A vs. D)*	<0.001* (n=230)*	<0.001 *(n=82)*	
TUG (seconds)			
Admission	8.7 (7.3 – 10.9)	7.2 (6.0 – 9.9)	0.002
Discharge	6.3 (5.0 – 7.5)	5.5 (4.5 – 6.8)	0.04
*p value (A vs. D)*	<0.001* (n=96)*	<0.001 *(n=45)*	
**Vitals**			
Heart rate (beats per minute)			
Admission	78 (±12)	80 (±13)	0.15
Discharge	78 (±13)	82 (±13)	0.26
*p value (A vs. D)*	0.87	0.67	
**Laboratory**			
Leukocytes (G/l)			
Admission	10.0 (8.1 – 11.9)	9.0 (7.3 – 11.3)	0.046
Discharge	6.9 (5.7 – 7.9)	6.5 (5.4 – 8.1)	0.27
*p value (A vs. D)*	<0.001	<0.001	
Hemoglobin (g/l)			
Admission	109 (99 – 120)	117 (106 – 132)	<0.001
Discharge	127 (117 – 136)	131 (132 – 141)	0.001
*p value (A vs. D)*	<0.001	<0.001	
Creatinine (µmol/l)			
Admission	86 (74 – 101)	83 (74 – 96)	0.16
Discharge	88 (75 – 100)	87 (75 – 100)	0.42
*p value (A vs. D)*	0.41	0.51	
eGFR (ml/min/1.7)			
Admission	80 (64 – 90)	81 (71 – 93)	0.18
Discharge	77 (63 – 91)	76 (67 – 90)	0.62
*p value (A vs. D)*	0.76	0.30	
CRP (mg/l)			
Admission	36 (23 – 66)	43 (26 – 71)	0.53
Discharge	5 (2 – 5)	5 (2 – 9)	0.75
*p value (A vs. D)*	<0.001	<0.001	
**Fitness at discharge (*n*=382)**			
Watt max	122 (97 – 160)	152 (110 – 188)	0.01
Watt max (%)	87 (69 – 103)	101 (83 – 113)	0.001
MET	6.7 (5.4 – 8.0)	8.0 (6.5 -8.9)	<0.001

Data are shown as mean and standard deviation or median and interquartile range if skewed. Categorical data are shown as numbers and percentages. *p* values are based on Kruskal Wallis test, ANOVA, chi square tests, or Fischer’s exact test as appropriate

*Abbreviations: 6MWT* 6-minute walk test, *A* admission, *CRP* C-reactive protein, *D* discharge, *eGFR* estimated glomerular filtration rate, *FIM* Functional independence measure, *HADS A* Hospital anxiety and depression scale for anxiety, *HADS D* Hospital anxiety and depression scale for depression, *HADS T* Hospital anxiety and depression scale total, *MNH* MacNew Heart, *TUG* Timed up and go test

### Fitness

At admission, patients who underwent MICS showed a significantly greater distance in the 6MWT (426 meters [IQR 336 – 483]) than patients who underwent OHS (381 meters [IQR 299 – 453]) (Table 2). At discharge, patients after MICS showed the best absolute fitness in exercise testing with an average of 101% of the maximum predicted watts (Table 2). Furthermore, both groups showed significant improvements in their 6MWT at discharge. However, in regression analyses improvements between OHS and MICS did not differ (β -5 (95% CI -26 – 14) (Table 7). In both groups, those who were under 60 years of age showed greater distance in the 6MWT at admission and at discharge (Tables 3 and 4). In regression analyses, the improvements in patients under 60 years of age remained significantly higher in univariable and fully adjusted models for OHS (β 46 (95% CI 26 – 68) and MICS (β 67 (95% CI 36 – 99) (Tables 8 and 9). Furthermore, within both groups, patients after revascularization and valve only surgery improved their 6MWT (Tables 5 and 6). However, in regression analyses, when comparing revascularization to valve surgery only and considering Bonferroni correction for multiple testing, neither improvements in the OHS group (β 6 (95% CI -14 – 25) nor in the in MICS group (β 51 (95% CI 7 – 96) remained significant (Tables 10 and 11).

**Table 3:** Changes in the 6-minute walk test score and quality of life measurements in patients after open heart surgery stratified by age

	**Age ≤ 60 years** ***n* = 75**	**Age > 60 years** ***n* = 225**	***p* value**
**Scores**			
6MWT (meters)			
Admission	421 (357 – 497)	363 (285 – 432)	<0.001
Discharge	562 (498 – 645)	510 (435 – 573)	<0.001
*p value (A vs. D)*	*<0.001*	*<0.001*	
FIM			
Admission	107 (93 – 116)	101 (90 – 112)	0.04
Discharge	124 (120 – 126)	124 (118 – 126)	0.39
*p value (A vs. D)*	*<0.001*	*<0.001*	
HADS A			
Admission	3 (1 – 6)	2 (1 – 5)	0.10
Discharge	2 (1 – 4)	1 (0 – 2)	<0.001
*p value (A vs. D)*	*0.003*	*<0.001*	
HADS D			
Admission	3 (1 – 7)	3 (1 – 5)	0.18
Discharge	1 (0 – 3)	1 (0 – 2)	0.02
*p value (A vs. D)*	*<0.001*	*<0.001*	
HADS T			
Admission	6 (3 – 12)	5 (2 – 10)	0.10
Discharge	3 (1 – 6)	1 (0 – 4)	<0.001
*p value (A vs. D)*	*<0.001*	*<0.001*	
MNH			
Admission	137 (117 – 151)	134 (121 – 149)	0.97
Discharge	161 (145 – 171)	160 (150 – 169)	0.86
*p value (A vs. D)*	*<0.001*	*<0.001*	
TUG (seconds)			
Admission	7.7 (6.2 – 8.6)	9.7 (8.1 – 12.0)	<0.001
Discharge	4.9 (4.2 – 6.5)	6.7 (5.8 – 8.4)	<0.001
*p value (A vs. D)*	*<0.001*	*<0.001*	

Data are shown as mean and standard deviation or median and interquartile range if skewed. *p* values are based on Kruskal Wallis test

*Abbreviations: 6MWT* 6-minute walk test, *CRP* C-reactive protein, *eGFR* estimated glomerular filtration rate, *FIM* Functional independence measure, *HADS A* Hospital anxiety and depression scale for anxiety, *HADS D* Hospital anxiety and depression scale for depression, *HADS T* Hospital anxiety and depression scale total, *MNH* MacNew Heart, *TUG* Timed up and go test

**Table 4:** Changes in the 6-minute walk test score and quality of life measurements in patients after minimally invasive cardiac surgery stratified by age

	**Age ≤ 60 years** ***n* = 38**	**Age > 60 years** ***n* = 65**	***p* value**
**Scores**			
6MWT (meters)			
Admission	443 (360 – 545)	415 (332 – 465)	0.11
Discharge	650 (570 – 706)	543 (448 – 614)	<0.001
*p value (A vs. D)*	*<0.001*	*<0.001*	
FIM			
Admission	111 (97 – 118)	106 (97 – 114)	0.18
Discharge	125 (122 – 126)	125 (121 – 126)	0.79
*p value (A vs. D)*	*<0.001*	*<0.001*	
HADS A			
Admission	3 (0 – 6)	3 (1 – 6)	0.97
Discharge	1 (0 – 3)	1 (0 – 2)	0.37
*p value (A vs. D)*	*<0.001*	*<0.001*	
HADS D			
Admission	2 (1 – 6)	3 (1 – 5)	0.99
Discharge	1 (0 – 1)	1 (0 – 2)	0.20
*p value (A vs. D)*	*0.001*	*<0.001*	
HADS T			
Admission	5 (1 – 11)	5 (3 – 10)	0.82
Discharge	2 (0 – 5)	2 (1 – 4)	0.96
*p value (A vs. D)*	*0.002*	*<0.001*	
MNH			
Admission	143 (133 – 154)	134 (123 – 144)	0.07
Discharge	167 (153 – 175)	165 (156 – 171)	0.34
*p value (A vs. D)*	*<0.001*	*<0.001*	
TUG (seconds)			
Admission	6.2 (5.6 – 9.9)	7.3 (6.5 – 10.0)	0.23
Discharge	5.0 (4.2 – 5.5)	6.3 (4.6 – 7.5)	0.02
*p value (A vs. D)*	*0.002*	*<0.001*	

Data are shown as mean and standard deviation or median and interquartile range if skewed. *p* values are based on Kruskal Wallis test

*Abbreviations: 6MWT* 6-minute walk test, *CRP* C-reactive protein, *eGFR* estimated glomerular filtration rate, *FIM* Functional independence measure, *HADS A* Hospital anxiety and depression scale for anxiety, *HADS D* Hospital anxiety and depression scale for depression, *HADS T* Hospital anxiety and depression scale total, *MNH* MacNew Heart, *TUG* Timed up and go test

**Table 5:** Changes in the 6-minute walk test score and quality of life measurements in patients after open heart surgery stratified by type of procedure

	**Revascularization** ***n* = 161**	**Valve** ***n* = 70**	***p* value**
**Scores**			
6MWT (meters)			
Admission	387 (295 – 476)	377 (314 – 450)	0.73
Discharge	519 (444 – 594)	535 (480 – 588)	0.22
*p value (A vs. D)*	*<0.001*	*<0.001*	
FIM			
Admission	101 (88 – 112)	107 (93 – 113)	0.12
Discharge	124 (118 – 126)	124 (120 – 126)	0.46
*p value (A vs. D)*	*<0.001*	*0.003*	
HADS A			
Admission	2 (1 – 5)	2 (1 – 5)	0.93
Discharge	1 (0 – 3)	1 (0 – 1)	0.33
*p value (A vs. D)*	*<0.001*	*<0.001*	
HADS D			
Admission	3 (1 – 6)	2 (0 – 4)	0.02
Discharge	1 (0 – 2)	1 (0 – 1)	0.33
*p value (A vs. D)*	*<0.001*	*0.005*	
HADS T			
Admission	5 (3 – 11)	5 (1 – 9)	0.19
Discharge	2 (1 – 5)	1 (0 – 3)	0.19
*p value (A vs. D)*	*<0.001*	*<0.001*	
MNH			
Admission	133 (120 – 148)	133 (125 – 149)	0.22
Discharge	158 (148 – 168)	163 (152 – 172)	0.18
*p value (A vs. D)*	*<0.001*	*0.001*	
TUG (seconds)			
Admission	8.7 (7.1 – 11.0)	8.7 (7.6 – 10.8)	0.92
Discharge	6.5 (5.2 – 7.8)	6.2 (4.9 – 7.1)	0.27
*p value (A vs. D)*	*<0.001*	*0.003*	

Data are shown as mean and standard deviation or median and interquartile range if skewed. *p* values are based on Kruskal Wallis test

*Abbreviations: 6MWT* 6-minute walk test, *CRP* C-reactive protein, *eGFR* estimated glomerular filtration rate, *FIM* Functional independence measure, *HADS A* Hospital anxiety and depression scale for anxiety, *HADS D* Hospital anxiety and depression scale for depression, *HADS T* Hospital anxiety and depression scale total, *MNH* MacNew Heart, *TUG* Timed up and go test

**Table 6:** Changes in the 6-minute walk test score and quality of life measurements in patients after minimally invasive cardiac surgery stratified by type of procedure

	**Revascularization** ***n* = 13**	**Valve** ***n* = 84**	***p* value**
**Scores**			
6MWT (meters)			
Admission	411 (342 – 471)	435 (335 – 493)	0.58
Discharge	525 (420 – 640)	593 (480 – 593)	0.14
*p value (A vs. D)*	*<0.001*	*<0.001*	
FIM			
Admission	113 (105 – 115)	107 (93 – 116)	0.37
Discharge	126 (122 – 126)	125 (121 – 126)	0.55
*p value (A vs. D)*	*<0.001*	*<0.001*	
HADS A			
Admission	4 (1 – 6)	3 (1 – 6)	0.54
Discharge	2 (0 – 3)	1 (0 – 2)	0.74
*p value (A vs. D)*	*<0.001*	*<0.001*	
HADS D			
Admission	2 (1 – 5)	2 (1 – 5)	0.89
Discharge	1 (0 – 2)	1 (0 – 2)	0.71
*p value (A vs. D)*	*<0.001*	*<0.001*	
HADS T			
Admission	5 (4 – 10)	5 (2 – 10)	0.78
Discharge	3 (0 – 5)	2 (1 – 4)	0.76
*p value (A vs. D)*	*<0.001*	*<0.001*	
MNH			
Admission	138 (119 – 157)	138 (124 – 152)	0.91
Discharge	171 (154 – 173)	165 (155 – 173)	0.37
*p value (A vs. D)*	*<0.001*	*<0.001*	
TUG (seconds)			
Admission	8.4 (6.7 – 9.4)	7.1 (5.9 – 10.5)	0.54
Discharge	6.9 (5.6 – 8.1)	5.2 (4.3 – 6.8)	0.13
*p value (A vs. D)*	*<0.001*	*<0.001*	

Data are shown as mean and standard deviation or median and interquartile range if skewed. *p* values are based on Kruskal Wallis test

*Abbreviations: 6MWT* 6-minute walk test, *CRP* C-reactive protein, *eGFR* estimated glomerular filtration rate, *FIM* Functional independence measure, *HADS A* Hospital anxiety and depression scale for anxiety, *HADS D* Hospital anxiety and depression scale for depression, *HADS T* Hospital anxiety and depression scale total, *MNH* MacNew Heart, *TUG* Timed up and go test

### QoL

At admission, the FIM and TUG score were better in the MICS groups compared to the OHS group (FIM 108 [IQR 97 – 116]; 102 [IQR 90 – 112], respectively and TUG 7.2 [IQR 6.0 – 9.9]; 8.7 [IQR 7.3 – 10.9], respectively). There were no significant differences in the other QoL measures (HADS A, HADS D, HADS T, and MNH score) among the two groups. During rehabilitation, we observed significant improvements in all QoL measures in all groups (Table 2) and subgroups (Tables 3, 4, 5 and 6). However, in regression analyses in fully adjusted models, these improvements were similar among the two groups and subgroups for all QoL measures (Tables 7, 8) Tables 9, 10 and 11. The only exception was the TUG score, where improvements were greater in patients under 60 years of age in the OHS group (β -1.2 (95% CI -1.8 – -0.5) (Table 8).

**Table 7:** Regression analysis for the association of fitness and multiple quality of life measures in patients with open heart surgery and minimally invasive surgery

	**Beta (95% CI), *p* value**						
**Model**	6MWT	FIM	HADS A	HADS D	HADS T	MNH	TUG
**1**	-15 (-33 – 4) *p* = 0.12	-0.6 (-2.0 – 0.8) *p* = 0.39	0.4 (-0.2 – 1.1) *p* = 0.21	0.2 (-0.4 – 0.8) *p* = 0.53	0.6 (-0.5 – 1.7) *p* = 0.29	-3.1 (-6.7 – 0.5) p = 0.09	0.1 (-0.5 – 0.7) *p* = 0.68
**2**	-13 (31 – 5) *p* = 0.14	-0.5 (-1.9 – 0.8) *p* = 0.45	0.5 (-0.2 – 1.1) *p* = 0.16	0.1 (-0.4 – 0.7) *p* = 0.64	0.6 (-0.5 – 1.7) *p* = 0.30	-3.1 (-6.7 – 0.6) *p* = 0.10	0.2 (-0.4 – 0.9) *p* = 0.50
**3**	-7 (-26 – 11) *p* = 0.44	-0.3 (-1.7 – 1.1) *p* = 0.69	0.2 (-0.5 – 1.0) *p* = 0.50	0.4 (-0.6 – 0.7) *p* = 0.90	0.3 (-0.9 – 1.5) *p* = 0.66	-3.6 (-7.1 – -0.2) *p* = 0.04	-0.1 (-0.7 – 0.5) *p* = 0.71
**4**	-5 (-26 – 14) *p* = 0.58	-0.3 (-1.7 – 1.1) *p* = 0.69	0.2 (-0.5 – 1.0) *p* = 0.50	0.4 (-0.6 – 0.7) *p* = 0.91	0.3 (-0.9 – 1.5) *p* = 0.67	-3.6 (-7.1 – -0.1) *p* = 0.04	-0.1 (-0.7 – 0.5) *p* = 0.80

Model 1 is corrected for 6MWT, FIM, HADS, MNH, or TUG at admission as appropriate

Model 2 includes all variables from Model 1 and is additionally corrected for sex (categorical) and age (continuous)

Model 3 includes all variables from Model 2 and is additionally corrected for fitness (maximum Watt (%))

Model 4 includes all variables from Model 3 and is additionally corrected for heart failure (categorical) and change in hemoglobin (continuous)

*Abbreviations 6MWT* 6-minute walk test, *FIM* Functional independence measure, *HADS A* Hospital anxiety and depression scale for anxiety, *HADS D* Hospital anxiety and depression scale for depression, *HADS T* Hospital anxiety and depression scale total, *MNH* MacNew Heart, *TUG* Timed up and go test

**Table 8:** Regression analysis for the association of fitness and multiple quality of life measures in patients under and over 60 years of age (after open heart surgery)

	**Beta (95% CI), *p* value**						
**Model**	6MWT	FIM	HADS A	HADS D	HADS T	MNH	TUG
**Univariate**	29 (7 – 52) *p* = 0.01	0.6 (-1.1– 2.4) *p* = 0.48	0.7 (0.0 – 1.4) *p* = 0.05	0.3 (-0.4 – 1.0) *p* = 0.37	1.0 (-0.3 – 2.2) *p* = 0.12	-0.5 (-4.7 – 3.7) *p* = 0.81	-1.1 (-1.8 - -0.4) *p* = 0.002
**Fully adjusted**	46 (26 – 68) *p* < 0.001	0.9 (-0.9 – 2.7) *p* = 0.30	0.7 (-0.1 – 1.6) *p* = 0.08	0.3 (-0.4 – 1.1) *p* = 0.39	1.0 (-0.4 – 2.4) *p* = 0.17)	-1.1 (-5.1 – 2.9) *p* = 0.60	-1.2 (-1.8 - -0.5) *p* = 0.001

Univariate model is corrected for 6MWT, FIM, HADS, MNH, or TUG at admission as appropriate

Fully adjusted model includes all variables from Model 1 and is additionally corrected for sex (categorical), fitness (maximum Watt (%)), heart failure (categorical), and change in hemoglobin (continuous)

**Table 9:** Regression analysis for the association of fitness and multiple quality of life measures in patients under and over 60 years of age (after minimally invasive cardiac surgery)

	**Beta (95% CI), *p* value**						
**Model**	6MWT	FIM	HADS A	HADS D	HADS T	MNH	TUG
**Univariate**	64 (29 – 100) *p* < 0.001	-0.2 (-2.3 – 1.8) *p* = 0.84	0.5 (-0.4 – 1.4) *p* = 0.24	-0.1 (-0.8 – 0.6) *p* = 0.75	0.4 (-0.9 – 1.8) *p* = 0.54	-3.7 (-9.4 – 2.0) p = 0.20	-0.6 (-1.4 – 0.2) *p* = 0.12
**Fully adjusted**	67 (36 – 99) *p* < 0.001	-1.1 (-3.2 – 1.0) *p* = 0.30	0.7 (-.4 – 1.7) *p* = 0.20	-0.1 (-0.9 – 0.7) *p* = 0.75	0.5 (-1.0 – 2.1) *p* = 0.50	-5.5 (-11.7 – 0.8) *p* = 0.08	-0.9 (-1.8 – 0.0) *p* = 0.06

Univariate model is corrected for 6MWT, FIM, HADS, MNH, or TUG at admission as appropriate

Fully adjusted model includes all variables from Model 1 and is additionally corrected for sex (categorical), fitness (maximum Watt (%)), heart failure (categorical), and change in hemoglobin (continuous)

*Abbreviations: 6MWT* 6-minute walk test, *FIM* Functional independence measure, *HADS A* Hospital anxiety and depression scale for anxiety, *HADS D* Hospital anxiety and depression scale for depression, *HADS T* Hospital anxiety and depression scale total, *MNH* MacNew Heart, *TUG* Timed up and go test

**Table 10:** Regression analysis for the association of fitness and multiple quality of life measures in patients with valve only surgery compared to revascularization only (after open heart surgery)

	**Beta (95% CI), *p* value**						
**Model**	6MWT	FIM	HADS A	HADS D	HADS T	MNH	TUG
**Univariate**	16 (-4 – 36) *p* = 0.12	0.8 (-0.9 – 2.4) *p* = 0.36	-0.6 (-1.4 – 0.1) *p* = 0.10	-0.1 (-0.8 – 0.5) *p* = 0.69	-0.7 (-1.9 – 0.6) *p* = 0.29	1.0(-2.9 – 5.0) *p* = 0.62	-0.6 (-1.3 – 0.1) *p* = 0.09
**Fully adjusted**	6 (-14 – 25) *p* = 0.56	0.4 (-1.4 – 2.1) *p* = 0.70	-0.5 (-1.4 – 0.4) *p* = 0.31	0.1(-0.7 – 0.9) *p* = 0.81	-0.3 (-1.8 – 1.2) *p* = 0.70	-0.7 (-4.9 – 3.4) *p* = 0.73	-0.3 (-1.0 – 0.5) *p* = 0.48

Univariate model is corrected for 6MWT, FIM, HADS, MNH, or TUG at admission as appropriate

Fully adjusted model includes all variables from Model 1 and is additionally corrected for sex (categorical), age (continuous), fitness (maximum Watt (%)), heart failure (categorical), and change in hemoglobin (continuous)

**Table 11:** Regression analysis for the association of fitness and multiple quality of life measures in patients with valve only surgery compared to revascularization only (after minimally invasive cardiac surgery)

	**Beta (95% CI), *p* value**						
**Model**	6MWT	FIM	HADS A	HADS D	HADS T	MNH	TUG
**Univariate**	58 (8 – 107) *p* = 0.02	-0.3 (-3.2 – 2.5) *p* = 0.81	0.2 (-1.1 – 1.5) *p* = 0.78	0.5 (-0.5 – 1.5) *p* = 0.30	0.73 (-1.2 – 2.7) *p* = 0.46	-1.9 (-9.9 – 6.1) *p* = 0.64	-1.2 (-2.4 – 0.0) *p* = 0.05
**Fully adjusted**	51 (7 – 96) *p* = 0.02	1.4 (-1.7 – 4.4) *p* = 0.37	-0.1 (-1.7 – 1.5) *p* = 0.92	0.3 (-1.0 – 1.5) *p* = 0.67	0.3 (-2.1 – 2.8) *p* = 0.78	-4.1 (-13.5 – 5.4) *p* = 0.40	-1.6 (-.3.0 - -0.1) *p* = 0.03

Univariate model is corrected for 6MWT, FIM, HADS, MNH, or TUG at admission as appropriate

Fully adjusted model includes all variables from Model 1 and is additionally corrected for sex (categorical), age (continuous), fitness (maximum Watt (%)), heart failure (categorical), and change in hemoglobin (continuous)

*Abbreviations: 6MWT* 6-minute walk test, *FIM* Functional independence measure, *HADS A* Hospital anxiety and depression scale for anxiety, *HADS D* Hospital anxiety and depression scale for depression, *HADS T* Hospital anxiety and depression scale total, *MNH* MacNew Heart, *TUG* Timed up and go test

## Discussion

To the best of our knowledge, for the first time, we demonstrated that improvements in terms of fitness during inpatient ECBR, as assessed by the 6MWT, did not differ between patients depending on the procedure for which they were referred. All patients significantly improved in the 6MWT and the improvements were equally large after OHS and MICS. Additionally, similar significant improvements in QoL measures were observed across all groups.

The 6MWT has been used for almost 40 years in patients with heart failure as a measure of exercise capacity and is a strong and independent predictor of morbidity and mortality [11–13]. Furthermore, exercise training improved the walking distance during the 6MWT in a heart failure population [14]. More recently, the use of the 6MWT has also been shown to be a valid and reliable method of assessing functional ability in EBCR and has become an integral part of fitness assessment [15–17]. Patients showed significantly greater improvements in walking distances after coronary artery bypass graft surgery if exercise training was included during rehabilitation [18] with equal improvements across all types of OHS [6]. However, to the best of our knowledge now previous study evaluated differences between OHS and MICS. We were able to show that improvements were similar for both groups, confirming that cardiac rehabilitation is important even for patients with only MICS. However, patients after OHS were slightly older and revascularization procedures were more common compared to patients after MICS, who more often underwent valvular procedures. Previous studies have shown that gains in fitness during outpatient cardiac rehabilitation are significantly larger in younger patient groups [19]. Our study is in line with these results and expands these results to inpatient cardiac rehabilitation, showing that patients under 60 years of age improved their 6MWT distance to larger degree than older patients after OHS as well as MICS.

Additionally, no previous study has analyzed if outcomes differ after revascularization or valve surgery only. Our results show that within the groups of OHS and MICS, patients show similar benefit in fitness after revascularization or valve surgery only, as assessed by the 6MWT. These results are encouraging and emphasize the importance of cardiac rehabilitation in a wide range of patient groups, especially since rehabilitation is still underutilized [2].

Since anxiety and depression are common among EBCR patients and significantly impact QoL and future cardiac events [20–22], another vital pillar of treatment during EBCR is psychological evaluation and support [7]. In fact, in addition to data from Cochrane reviews, two studies in which EBCR was supplemented by psychotherapy have recently been shown to reduce the HADS scores, improve QoL and adherence to EBCR, and reduced cardiovascular readmissions [9, 23, 24]. However, the interventions from these studies lasted from a minimum of 5 weeks to multiple months. The duration of psychotherapy in our study was only 21 days. Furthermore, the average HADS score was much greater in the previously mentioned studies than in our study. Nonetheless, a clinically meaningful improvement was still observed in our cohort across both groups. To the best of our knowledge, we are the first to stratify groups according to OHS and MICSand compare not only the HADS score but also multiple QoL outcomes, including the MNH, FIM, and TUG test, all of which have been validated previously for EBCR [25–28]. In our cohort, we demonstrated significant improvements in each measure from admission to discharge in both groups, as well in all subgroups. These groups differed significantly regarding heart failure, revascularization and valvular procedures, and the timing of the procedure. Interestingly, however, there was no difference in the degree of improvement for any score across the three groups during inpatient EBCR. These results may indicate that anxiety, depression, and reduced QoL might not be specific to a certain cardiovascular disease or procedure but rather represent a consequence of having a cardiovascular disease and procedure in general.

## Strengths and limitations

The strengths of this study lie in its prospective design and real-world setting, which involved comparing improvements during EBCR between different groups of patients who all completed a similar rehabilitation program.

However, due to the nature of admission to an inpatient ECBR, which is only possible directly from an acute care hospital, large differences in time between the procedure and admission are unlikely. Due to the observational cohort study design, residual confounding and selection biases remain, and hence, no causal inferences can be drawn. Although the effect of different patient characteristics has been considered in regression analyses, unknown factors remain. As most patients were of Caucasian descent, generalizability to other groups is limited. Additionally, due to the low number of female patients, who are unfortunately referred for EBCR less frequently [29], no sex-specific analysis was carried out. Furthermore, while we observed significant short-term improvements, it is not clear whether these improvements translate into meaningfully different long-term outcomes. Further long-term follow-up is warranted to investigate whether the shorter interventions during inpatient EBCR show similar benefits to the proven long-term benefits during outpatient EBCR. Finally, cardio-pulmonary exercise testing would have been ideal to compare fitness levels at referral and discharge. However, since many patients were not fit enough when starting EBCR so closely after surgery, a maximal exercise test was not feasible.

## Conclusion

In this real-world sample of inpatient EBCR patients, improvements in fitness, as assessed by the 6MWT, were similar after OHS and MICS. Furthermore, multiple QoL measures improved equally across both groups. These are encouraging results and emphasize the importance of EBCR. Additionally, the minimally invasive nature of the procedure and type of surgery should not deter referral to inpatient EBCR.

## Data Availability

The datasets used and/or analyzed during the current study are available from the corresponding author on reasonable request.

## Abbreviations

6MWT: 6-minute Walk Test
ANCOVA: Analysis of Covariance
CRP: C-reactive protein
EBCR: Exercise-Based Cardiac Rehabilitation
FIM: Functional Independence Measure
HADS: Hospital Anxiety and Depression Scale
HFpEF: Heart Failure with Preserved Ejection Fraction
HFmrEF: Heart Failure with Mid-Range Ejection Fraction
HFrEF: Heart Failure with Reduced Ejection Fraction
HFimpEF: Heart Failure with Improved Ejection Fraction
IQR: Interquartile Range
MI: Myocardial Infarction
MICS: Minimally Invasive Cardiac Surgery
MNH: Mac New Heart
OHS: Open Heart Surgery
TUG: Timed-Up and Go
QoL: Quality of Life

## References

[1] Dibben GO, Faulkner J, Oldridge N, Rees K, Thompson DR, Zwisler AD (2023). Exercise-based cardiac rehabilitation for coronary heart disease: a meta-analysis. European Heart Journal..

[2] Eijsvogels TMH, Maessen MFH, Bakker EA, Meindersma EP, Van Gorp N, Pijnenburg N (2020). Association of Cardiac Rehabilitation With All-Cause Mortality Among Patients With Cardiovascular Disease in the Netherlands. JAMA Netw Open..

[3] Kleczynski P, Trebacz J, Stapor M, Sobczynski R, Konstanty-Kalandyk J, Kapelak B (2021). Inpatient Cardiac Rehabilitation after Transcatheter Aortic Valve Replacement Is Associated with Improved Clinical Performance and Quality of Life. JCM..

[4] Schmidt T, Kowalski M, Bjarnason-Wehrens B, Ritter F, Mönnig G, Reiss N (2022). Feasibility of inpatient cardiac rehabilitation after percutaneous mitral valve reconstruction using clipping procedures: a retrospective analysis. BMC Sports Sci Med Rehabil..

[5] Salzwedel A, Nosper M, Röhrig B, Linck-Eleftheriadis S, Strandt G, Völler H (2014). Outcome quality of in-patient cardiac rehabilitation in elderly patients – identification of relevant parameters. Eur J Prev Cardiolog..

[6] Fiorina C, Vizzardi E, Lorusso R, Maggio M, De Cicco G, Nodari S (2007). The 6-min walking test early after cardiac surgery Reference values and the effects of rehabilitation programme. Eur J Cardio-Thoracic Surg.

[7] Piepoli MF, Corrà U, Adamopoulos S, Benzer W, Bjarnason-Wehrens B, Cupples M, et al. Secondary prevention in the clinical management of patients with cardiovascular diseases. Core components, standards and outcome measures for referral and delivery: A Policy Statement from the Cardiac Rehabilitation Section of the European Association for Cardiovascular Prevention & Rehabilitation. Endorsed by the Committee for Practice Guidelines of the European Society of Cardiology. Eur J Prev Cardiolog. 2014;21(6):664–81. 10.1177/2047487312449597 22718797

[8] Visseren FLJ, Mach F, Smulders YM, Carballo D, Koskinas KC, Bäck M (2021). 2021 ESC Guidelines on cardiovascular disease prevention in clinical practice. Eur Heart J..

[9] Holdgaard A, Eckhardt-Hansen C, Lassen CF, Kjesbu IE, Dall CH, Michaelsen KL (2023). Cognitive-behavioural therapy reduces psychological distress in younger patients with cardiac disease: a randomized trial. Eur Heart J.

[10] Abreu A, Schmid JP, Piepoli M, editors. ESC Handbook of Cardiovascular Rehabilitation: A practical clinical guide [Internet]. 1st ed. Oxford University Press; 2020 [cited 2024 May 1]. Available from: https://academic.oup.com/esc/book/31780.

[11] Guyatt GH, Sullivan MJ, Thompson PJ, Fallen EL, Pugsley SO, Taylor DW (1985). The 6-minute walk: a new measure of exercise capacity in patients with chronic heart failure. Can Med Assoc J..

[12] Bittner V, Weiner DH, Yusuf S, Rogers WJ, McIntyre KM, Bangdiwala SI (1993). Prediction of mortality and morbidity with a 6-minute walk test in patients with left ventricular dysfunction. SOLVD Investigators. JAMA..

[13] Willenheimer R, Erhardt LR (2000). Value of 6-min-walk test for assessment of severity and prognosis of heart failure. The Lancet..

[14] Meyer K, Schwaibolda M, Westbrook S, Beneke R, Hajric R, Lehmann M (1997). Effects of exercise training and activity restriction on 6-minute walking test performance in patients with chronic heart failure. Am Heart J.

[15] Hamilton DM, Haennel RG (2000). Validity and Reliability of the 6-Minute Walk Test in a Cardiac Rehabilitation Population. JCardiopulm Rehabil..

[16] Bellet RN, Adams L, Morris NR (2012). The 6-minute walk test in outpatient cardiac rehabilitation: validity, reliability and responsiveness—a systematic review. Physiotherapy..

[17] Chen YC, Chen KC, Lu LH, Wu YL, Lai TJ, Wang CH (2018). Validating the 6-minute walk test as an indicator of recovery in patients undergoing cardiac surgery: A prospective cohort study. Medicine.

[18] Zanini M, Nery RM, De Lima JB, Buhler RP, Da Silveira AD, Stein R (2019). Effects of Different Rehabilitation Protocols in Inpatient Cardiac Rehabilitation After Coronary Artery Bypass Graft Surgery: A RANDOMIZED CLINICAL TRIAL. J Cardiopulm Rehabi Prev.

[19] Sandercock G, Hurtado V, Cardoso F (2013). Changes in cardiorespiratory fitness in cardiac rehabilitation patients: A meta-analysis. I J Cardiol.

[20] Von Känel R, Rosselet K, Gessler K, Haeussler A, Aschmann J, Rodriguez H (2022). Preoperative depression and anxiety as predictors of postoperative C-reactive protein levels in patients undergoing cardiac surgery: a prospective observational study. Swiss Med Wkly..

[21] Meijer A, Conradi HJ, Bos EH, Thombs BD, Van Melle JP, De Jonge P (2011). Prognostic association of depression following myocardial infarction with mortality and cardiovascular events: a meta-analysis of 25 years of research. Gen Hosp Psychiatry.

[22] Palacios J, Khondoker M, Mann A, Tylee A, Hotopf M (2018). Depression and anxiety symptom trajectories in coronary heart disease: Associations with measures of disability and impact on 3-year health care costs. J Psychosom Res.

[23] Wells A, Reeves D, Capobianco L, Heal C, Davies L, Heagerty A (2021). Improving the Effectiveness of Psychological Interventions for Depression and Anxiety in Cardiac Rehabilitation: PATHWAY—A Single-Blind, Parallel, Randomized, Controlled Trial of Group Metacognitive Therapy. Circulation..

[24] Richards SH, Anderson L, Jenkinson CE, Whalley B, Rees K, Davies P, et al. Psychological interventions for coronary heart disease. Cochrane Heart Group, editor. Cochrane Database of Systematic Reviews. 2017 Apr 28 [cited 2024 Jan 24];2021(2). Available from: http://doi.wiley.com/10.1002/14651858.CD002902.pub4. 10.1002/14651858.CD002902.pub4 PMC6478177 28452408

[25] Höfer S, Lim L, Guyatt G, Oldridge N. The MacNew Heart Disease health-related quality of life instrument: a summary. Health Qual Life Outcomes. 2004;2:3. 10.1186/1477-7525-2-3. Published 2004 Jan 8. 10.1186/1477-7525-2-3 PMC341459 14713315

[26] Maes S, De Gucht V, Goud R, Hellemans I, Peek N (2008). Is the MacNew quality of life questionnaire a useful diagnostic and evaluation instrument for cardiac rehabilitation?. Eur J Cardiovasc Prev Rehabil.

[27] Sasanuma N, Takahashi K, Itani Y, Tanaka T, Yamauchi S, Mabuchi S (2015). Motor and cognitive function analysis for home discharge using the Functional Independence Measure in patients undergoing cardiac rehabilitation at a long-term acute-care hospital. Eur J Phys Rehabil Med..

[28] Bellet RN, Francis RL, Jacob JS, Healy KM, Bartlett HJ, Adams L (2013). Timed Up and Go Tests in Cardiac Rehabilitation: RELIABILITY AND COMPARISON WITH THE 6-MINUTE WALK TEST. J Cardiopulm Rehabili Prev.

[29] Patel N, Ahmad M. Correspondence to the European Heart Journal—Quality of Care and Clinical Outcomes in response to the paper by Thygesen et al. European Heart Journal - Quality of Care and Clinical Outcomes. 2022;8(4):478. 10.1093/ehjqcco/qcac003 35030238

